# On the Rise of Slurry Electrolysis for Energy Applications

**DOI:** 10.1002/asia.202500851

**Published:** 2025-09-15

**Authors:** Jingjing Xiong, Guanwu Lian, Zhongxin Chen

**Affiliations:** ^1^ Guangdong Basic Research Center of Excellence for Aggregate Science School of Science and Engineering The Chinese University of Hong Kong Shenzhen 518172 China

**Keywords:** Electro‐percolation network, Flow electrolyzer, Fluidized electrolysis, Semi‐solid flow batteries, Slurry electrolysis

## Abstract

Slurry electrolysis represents a paradigm shift in diverse electrochemical applications by replacing conventional fixed electrodes with flowable suspensions of electrochemically active particles for scalable energy conversion and storage. In this perspective, we will detail the recent advances of slurry electrolysis in electrocatalysis, semi‐solid flow batteries, and flow‐electrode capacitive deionization, featuring the fundamental role of the formation of the electro‐percolation network in charge transport in these slurry systems. Mechanistic insights into the dynamic evolution of flowable suspension are provided via advanced characterization techniques, including operando EIS, computational fluid dynamics calculations, and in situ imaging. Finally, we will provide a forward‐looking perspective in tackling the challenges in electrochemical side reactions and slurry instability for their practical applications.

## Graphical abstract

In this study, we will provide a forward‐looking perspective of slurry electrolysis, featuring its diverse application across electrocatalysis, energy storage, and water desalination, as well as the critical role of the electro‐percolation network in charge transport.

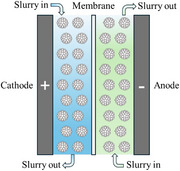

## 1 Introduction

Conventional electrochemical systems are built with fixed‐electrode architectures, which are permanently positioned within the electrochemical cell to facilitate charge transfer and electrochemical reactions.^[^ ^1^ ^]^ Despite significant advances, these fixed‐electrode systems are usually constrained by limited mass transport and degraded electrode performance. Such problems become more pronounced in applications demanding high throughput, long‐term stability, and dynamic compositional control in future energy transformations.^[^ ^2^, ^3^ ^]^

To this end, the employment of flowable suspensions of electrochemically active particles in slurry electrolysis represents a paradigm shift in electrochemical designs, which allows the continuous electrode circulation, enhanced interfacial activity, and on‐stream adjustments.^[^ ^2^, ^3^ ^]^ Such a concept has been actively applied across multiple areas in electrocatalysis, semi‐solid flow batteries (SSFBs), and flow‐electrode capacitive deionization (FCDI).^[^ ^4^, ^5^, ^6^, ^7^, ^8^ ^]^ While these technologies serve distinct purposes, ranging from energy conversion and storage to water desalination, they all benefit from the intrinsic advantages of slurry electrolysis. For instance, the continuous flow and renewal of catalyst particles could significantly enhance the exposure of active sites and improve gas evolution dynamics in gas‐involving reactions.^[^ ^5^, ^9^, ^10^ ^]^ Meanwhile, the use of highly concentrated suspensions of redox‐active materials in SSFBs allows for high volumetric energy densities and scalable system architectures.^[^ ^8^ ^]^ The fast charging/discharging operations, along with decoupling of energy and power outputs, enable the slurry flow electrode to support grid‐scale energy/power ratings.^[^ ^11^ ^]^ Similarly, the ability to continuously regenerate flowing carbon electrodes eliminates capacity limitations associated with static electrode systems to improve the desalination efficiency in FCDIs.^[^ ^7^ ^]^

From a fundamental point of view, the performance of slurry systems, regardless of the specific applications, is governed by intermittent contacts between particles and between particles and current collectors.^[^ ^4^, ^12^ ^]^ This results in the formation of a transient and evolving conductive network (electro‐percolation network) that is strongly correlated to the particle concentration. This is distinct from the well‐defined electron pathways in conventional fixed‐electrode systems, thus leading to higher technical complexities in optimizing the slurry systems.^[^ ^3^ ^]^ Various experimental and computational techniques have been employed in understanding the formation mechanism.^[^ ^3^, ^13^, ^14^ ^]^ Meanwhile, technical challenges also remain in the instability of slurry (particle settling and agglomeration) and performance fluctuation due to its evolving structure, which urges the development of a comprehensive toolkit in measuring the rheological behavior, mass transfer kinetics, and visualizing the dynamic movement of particles inside the electrolyzer.^[^ ^13^, ^15^, ^16^ ^]^

This perspective provides a forward‐looking discussion of slurry electrolysis, focusing on its distinctive flowable particulate features, the critical role of the electro‐percolation network, as well as advanced characterization techniques in monitoring the dynamic structural evolution across diverse applications. Possible solutions toward challenges in electrochemical stability, material design, and scalable deployment will also be discussed.

## 2 Diverse Applications in Energy Conversion and Storage

Enjoying the benefits of flowable suspensions with dynamic electrochemical responses, the application of slurry electrolysis has spread from catalytic systems to transformative energy storage and water desalination.

**Figure 1:**
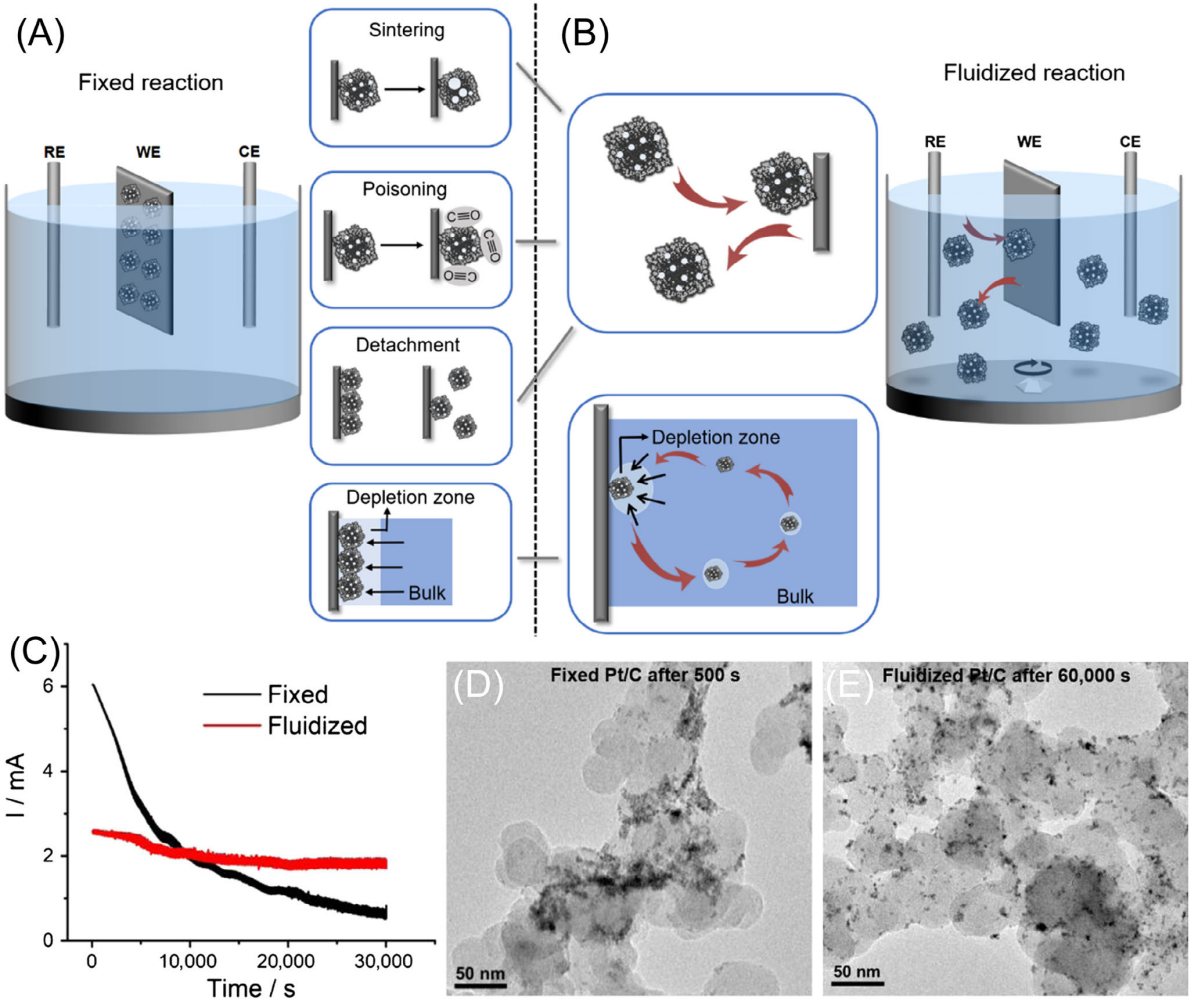
Concept of slurry electrolysis. A) Comparison between fixed‐electrode and B) slurry (fluidized) electrocatalysis; C) current outputs of the Pt/C‐catalyzed OER under fixed‐electrode and fluidized conditions; D) TEM images of the Pt nanoparticles on the fixed Pt/C after only 500 seconds of OER, and E) those on the fluidized Pt/C after 60,000 seconds. Reproduced with permission from Ref. [5].

A typical example is slurry electrocatalysis (or fluidized electrocatalysis) in Figure 1. Unlike conventional fixed electrodes with immobilized catalysts using binders and conductive additives, fluidized systems allow suspended catalyst particles to engage with the electrode through stochastic collisions, thus reducing continuous electrochemical stress and mitigating unwanted sintering and surface poisoning.^[^ ^5^, ^17^ ^]^ This intermittent interaction is particularly beneficial for addressing the problem of compromised catalyst stability in the oxygen evolution reaction (OER). As shown in  Figure 1C–E, a fixed Pt/C electrode undergoes a rapid decay in current density within the first 500 seconds under high‐potential conditions. In contrast, a fluidized Pt/C system could maintain a stable current for over 60000 seconds. This is further confirmed by the severe degradation of the spent, fixed Pt/C particles in the TEM analysis, while the fluidized sample remains well‐dispersed and intact even after extended operation.^[^ ^5^ ^]^ Beyond the OER, fluidized electrocatalysis is also applicable in selective organic transformations, such as the methanol oxidation reaction and the electrocatalytic hydrogenation of furfural using nanoparticle catalysts.^[^ ^18^ ^]^ Enhanced selectivity could be seen in these reactions due to suppressed competing side reactions (e.g., hydrogen evolution) by fluidization. Meanwhile, the dynamic movement of particulate electrodes further enhances mass transport for uniform reactant supply and efficient product desorption. This is crucial for reactions involving poorly soluble or slow‐diffusing organic molecules.

**Figure 2:**
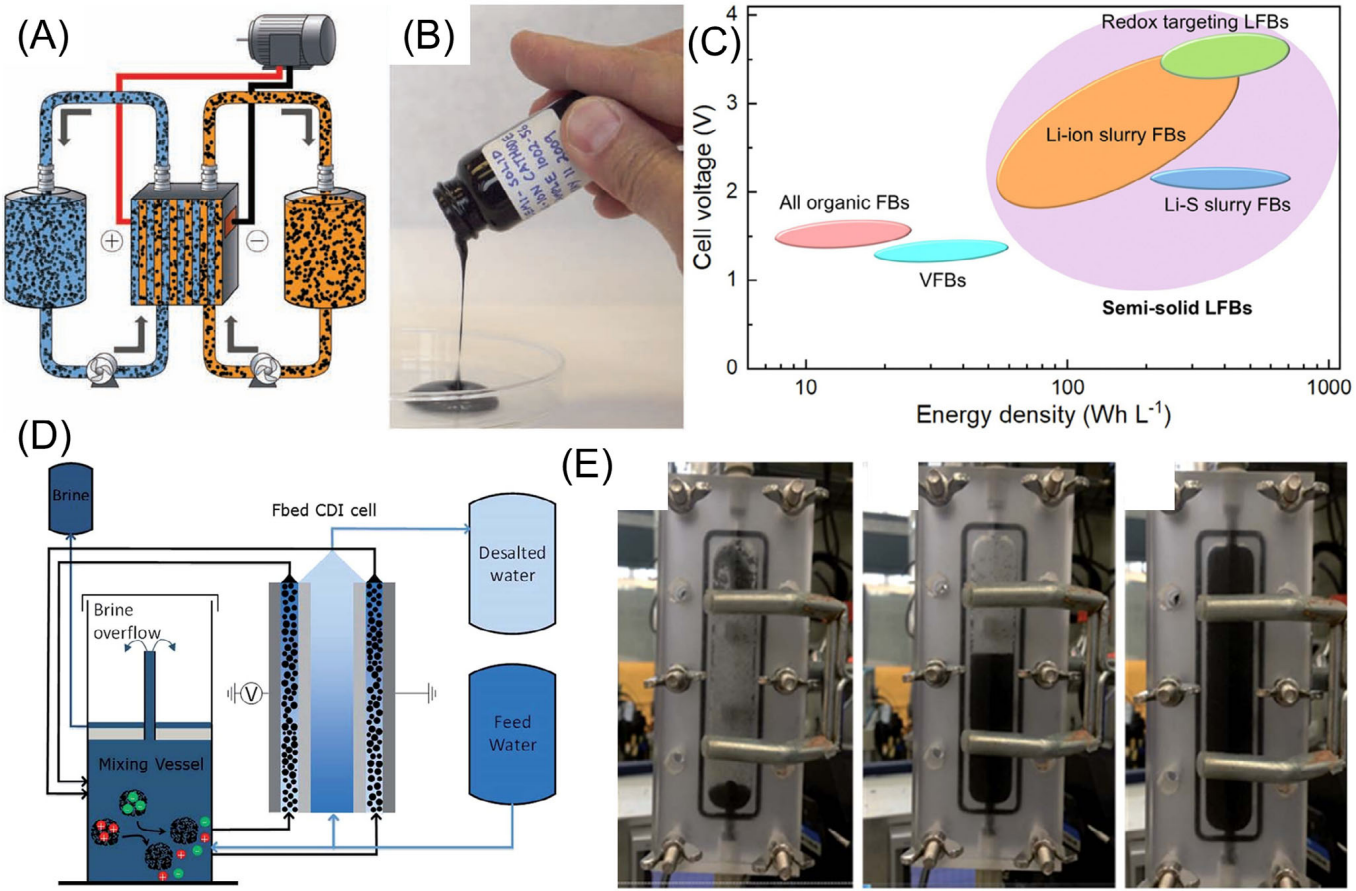
Application in semi‐solid flow batteries and capacitive deionisation. A) Semi‐solid flow batteries using flowing lithium‐ion cathode and anode suspensions; B) fluid semi‐solid suspension containing LiCoO_2_ powder as the active material and Ketjen black as the dispersed conductive phase, dispersed in alkyl carbonate electrolyte; C) cell voltage versus energy density of various flow batteries; D) schematic of the fluidized bed capacitive deionization system for continuous desalination and closed‐loop electrode regeneration and re‐use; E) pictures of the rise of the fluidized bed in the optically accessible compartment, and are sequential in time. A), B) Reprinted with permission from Ref. [8]. C) Reprinted with permission from Ref. [4]; D), E) Reprinted with permission from Ref. [22].

In the context of energy storage, flowable electrodes composed of highly concentrated redox‐active materials in a liquid electrolyte enable the independent scaling of energy and power density in semi‐solid flow batteries (SSFBs) in Figure 2A.^[^ ^4^, ^8^, ^19^, ^20^ ^]^ This is superior to conventional batteries, where energy and power are inherently linked through fixed electrode geometry. A representative SSFB formulation includes LiCoO_2_ as the active material and Ketjen black as a dispersed conductive phase in an alkyl carbonate‐based electrolyte in Figure 2B, ensuring high energy density and sufficient electronic conductivity for stable and reversible charge‐discharge cycling.^[^ ^8^ ^]^ Such a design combines the high energy density of lithium‐ion batteries with the modular scalability of flow architectures. By breaking through the solubility limit in vanadium and all organic redox flow batteries, SSFBs demonstrate superior energy density, as shown in the comparison figure in Figure 2C.^[^ ^4^ ^]^ Recent advances related to material design, advanced diagnostics and new applications are also documented.^[^ ^21^ ^]^ Likewise, the same principle could be extended to water desalination through flow‐electrode capacitive deionization (FCDI) in Figure 2D.^[^ ^22^ ^]^ During continuous electrode circulation of carbon electrodes in the desalination cell, salt ions are electro‐adsorbed onto the electrode surface via electrical double‐layer (EDL) formation. This dynamic electrode configuration overcomes the saturation limitations of static capacitive deionization, leading to long‐term and uninterrupted desalination.^[^ ^6^, ^7^ ^]^ The electrode suspension dynamics of FCDI are further visualized in the optical compartments in Figure 2E. This offers valuable insights into particle distribution, electrode–electrolyte contact, and mass transport behavior for understanding the correlation between carbon weight percentage and flow electrolyzer performance in varying conditions.^[^ ^22^ ^]^ Under the optimized condition using a mixed carbon slurry, a stable average salt removal rate of 4.41 µmol cm^−2^ min^−1^ could be achieved with a charging efficiency of ∼96% and molar energy consumption of 0.043 kWh mol^−1^.^[^ ^13^ ^]^ A recent report by Wu et al. also developed a membrane‐current collector (MCC) configuration for practical and scale‐up desalination. Such a configuration shortens the charge‐transfer distance between the membrane and electrode and simplifies the stack configuration in FCDIs.^[^ ^23^ ^]^

Meanwhile, we should also point out the critical difference among fluidized electrocatalysis, SSFBs, and FCDIs in the working principle, the electro‐percolation network, and rheological behaviors.^[^ ^3^, ^5^, ^6^ ^]^ Faradaic reactions occur in both fluidized electrocatalysis and SSFBs, urging for a delicate design of the electrode material to facilitate the sluggish electrochemical reaction(s). However, FCDIs are based on non‐faradaic processes (capacitive collision), where maximizing ion‐accessible surface and preventing ohmic loss are crucial for an energy‐efficient operation. Regarding the electro‐percolation network formation, fluidized electrocatalysis usually involve transient (intermittent) contact between catalyst particles and the electrode, particularly when operating in a dilute to semi‐concentrated regime. This requires a fine‐tuning on the collision frequency and bubble management to promote the electron transport.^[^ ^5^ ^]^ In contrast, both SSFBs and FCDIs can be operated in concentrated regime above the percolation threshold, where a persistent conductive network will be formed. This also reflects in the different rheological behaviors of the electrolyte among these applications. Non‐Newtonian behavior governs in the dense, percolated paste in SSFBs, while the dilute slurry exhibits near‐Newtonian to shear‐thinning behavior in the fluidized electrocatalysis.^[^ ^4^ ^]^ In this regard, a unified framework that leverages the dynamic nature of particulate electrodes across diverse applications could be established for enhancing the electrochemical performance in slurry electrolysis.^[^ ^4^ ^]^

## 3 Formation and Monitoring of the Electro‐Percolation Network

As mentioned above, the formation of a transient and evolving conductive network via the intermittent contacts between flowing particles is a unique feature of slurry electrolysis. Hence, it is obvious that the density of particles (or solid content) will have a profound influence on the network formation.^[^ ^4^, ^24^ ^]^ Below a certain limit, the inter‐particle collision is insufficient to trigger the formation of a continuous conductive path across the electrode gap, leading to an “insulator” behavior of the slurry system. This minimum particle concentration is known as the percolation threshold, where a 3D, global conductive network will only emerge above this limit.^[^ ^12^ ^]^

**Figure 3:**
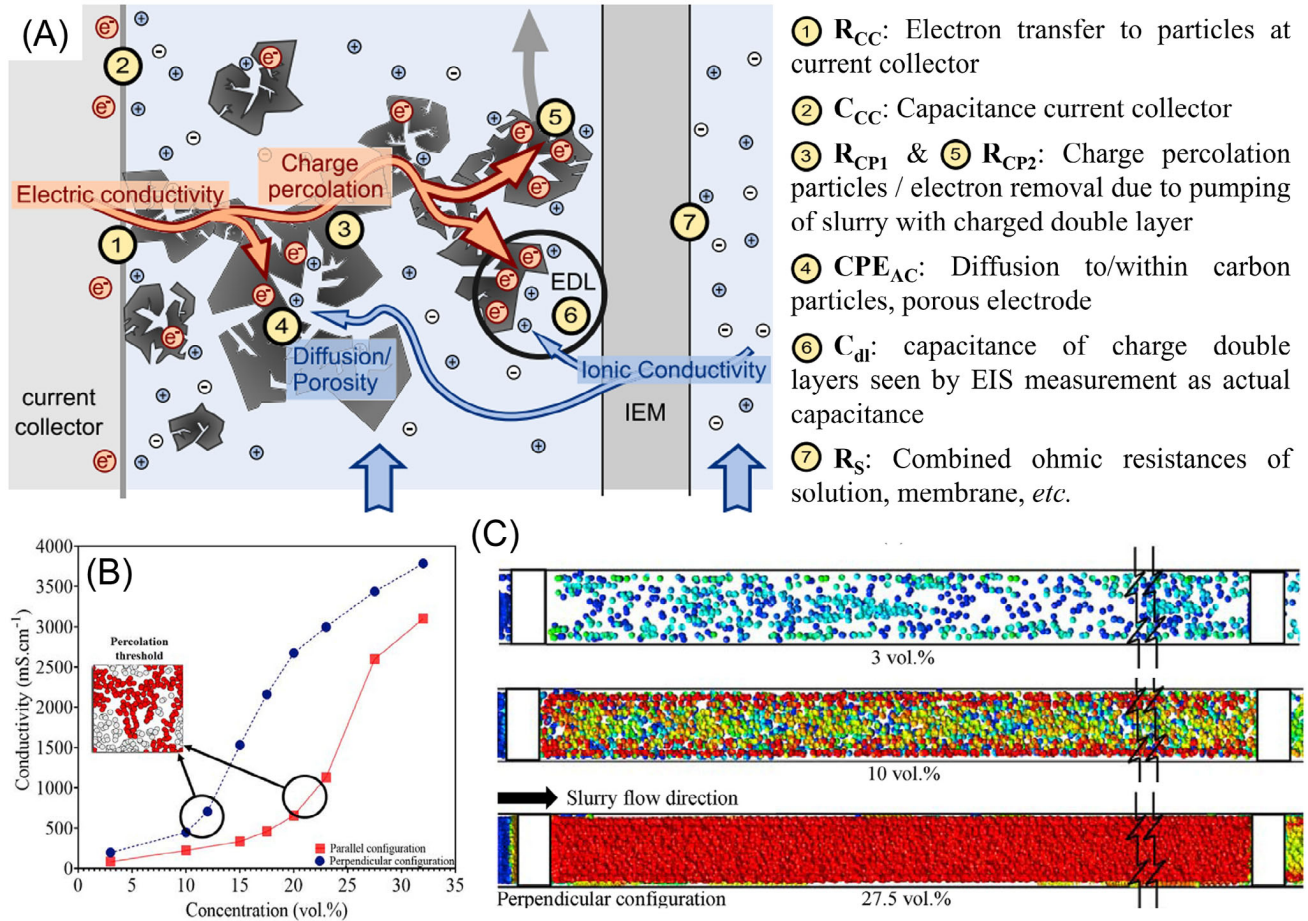
Mechanism of electron transport. A) The formation of electro‐percolation networks in the slurry system, showing the effective electron pathway is dominated by particle collision; B) comparison of the percolation threshold for perpendicular and parallel configurations at Re = 1 × 10^−3^; C) charge distribution around working electrode (right) and counter electrode (left) in perpendicular configuration in the mid‐section of the channel for slurry electrodes with the different concentrations of 43.2, 18.2, and 5.8 wt% (27.5, 10, and 3 vol%, respectively) using perpendicular current collector configurations at Re = 1 × 10^−3^. Percolation threshold is defined as the critical concentration of conductive particles needed to form a continuous network for efficient electron transport. a) Adapted with permission from Ref. [12]. B), C) Reprinted with permission from Ref. [14].

An in‐depth explanation of the electro‐percolation network is provided in Figure 3A, which represents (1) the electron transfer at the current collector (R_CC_ and C_CC_), (2) the influence of diffusion and electrode porosity (R_CP1_ and CPE_AC_), (3) the capacitance and current induced by convection (R_CP2_ and C_dl_), and (4) the combined, serial resistance of solutions, membranes, etc. (R_S_).^[^ ^12^ ^]^ With increasing solid contents, the most affected factors are the capacitance changes at the current collector (due to direct contact between carbon particles with the current collector, C_CC_), and the capacitance and current induced by convection (R_CP2_, charge percolation resistance, and C_dl_, capacitance of charge double layers). It was also found that C_dl_ increases slowly at low activated carbon contents and suddenly increases above 8 wt% of carbon content. This is attributed to an ineffective charge percolation, resulting in an incomplete use of the maximum adsorption capacity, as not all carbon particles are charged. In contrast, the capacitance increases linearly when reaching the percolation limit due to an effective charge percolation.^[^ ^12^ ^]^

Apart from the solid contents, the transition across the percolation limit is also influenced by the shape of the flowable particles and the electrode geometry. As shown in Figure 3B, Rosengarte et al. conducted a comprehensive study on the effect of current collector shapes and configurations on the percolation threshold.^[^ ^25^ ^]^ With a perpendicular configuration of the current collector, the conductivity of slurry electrodes doubles as the velocity increases almost three orders of magnitude, while it decreases by approximately 50% over the same velocity range in a traditional parallel configuration. This leads to a much lower percolation limit of 21.5 wt% (12 vol%) in the perpendicular setting than 33.4 wt% (20 vol%) in parallel.^[^ ^14^ ^]^ Likewise, introducing multi‐dimensional‐sized nanoparticles (e.g., the tubular carbon nanotubes and spherical carbon black particles) will enhance contact efficiency by forming a point‐to‐line‐to‐surface connection morphology. This effectively guarantees a robust charge electro‐percolation network with diverse pathways and facilitates the charge transfer.^[^ ^13^, ^26^ ^]^

In this regard, multiphase flow simulations are vital for understanding the coupled phenomena of fluid flow with charge transport as a function of the volume fraction, material, size, and shape of the particles and the fluid flow regime.^[^ ^24^, ^27^ ^]^ A common approach would be the coupling of computational fluid dynamics with the discrete element method (CFD‐DEM), which is a type of the Eulerian–Lagrangian method that calculates information such as particle trajectories and considers the complexity of particle‐particle interactions in the charge transfer process.^[^ ^14^, ^27^ ^]^ As shown in Figure 3C, a well‐developed conductive network with densely populated charged particles is visible at a high solid content of 43.2 wt% (27.5 vol%) in the CFD‐DEM calculations. This offers a relatively uniform charge distribution across the working and counter electrodes and much higher current densities in the slurry system. The conductive network becomes increasingly fragmented at lower solid contents of 5.8 ∼ 18.2 wt% (3 ∼ 10 vol%), leading to localized charge accumulation near the current collectors and regions of poor conductivity within the bulk suspension. These inhomogeneities raise local resistance and limit the electrode utilization, thus reducing the device performance.^[^ ^14^ ^]^ Notwithstanding these insights, the particle–fluid and particle–particle interactions in current CFD‐DEM modeling are often over‐simplified by using assumptions of Newtonian carrier, smooth non‐porous spheres, and near‐contact hydrodynamics, while charge transfer is also treated as instantaneous or by averaging upon collision.^[^ ^14^, ^24^, ^27^ ^]^ This significantly differs from the complex particle collisons, polydispersity of porous particles, and the non‐Newtonian rheology of the slurry. According to Lohaus et al.,^[^ ^24^ ^]^ mean‐field assumptions are usually invalid as only a subset of particles participate in charge transfer and a percolation threshold governs current rise. A practical remedy is to make charge transfer resistance‐limited. For instance, Heidarian et al. revealed the dominance of particle‐collector contacts via incorporating Brownian motion and introducing a charge transfer efficiency coefficient based on collision time and contact area.^[^ ^14^ ^]^ A compromise has to be made between the accuracy of modeling and computational cost, where open questions remain in the structural dynamism of electro‐percolation network under shearing and external stimuli, as well as upscaling to cell and stack levels.

We should also point out the influence of external forces, such as shear and electric fields, on the dynamic response of the conductive network. For instance, applying a shear rate above the percolation concentration in shear‐thinning fluids could lead to the formation of agglomerates and the breakdown of the conductive network.^[^ ^11^ ^]^ The application of an external electric field may counteract this disruption by aligning particles along the electric field.^[^ ^3^, ^4^ ^]^ This is crucial for semi‐solid flow batteries to maintain a stable electro‐percolation network for high energy densities and long cycle life. Likewise, such an alignment of conductive materials could be achieved by external flow field and magnetic field, thus enabling real‐time modulation of the percolation threshold across the electrode‐electrolyte interface.^[^ ^28^, ^29^ ^]^ To this end, a more in‐depth discussion on the use of solid particles in flow chemistry could be found in the reviews by J. Yue and H. L. D. Hayes, which tackle the pump design for non‐Newtonian fluids, reactor design to prevent clogging and particle settling, heat management, and strategies for ensuring uniform flow distribution in large‐scale systems.^[^ ^30^, ^31^ ^]^

**Figure 4:**
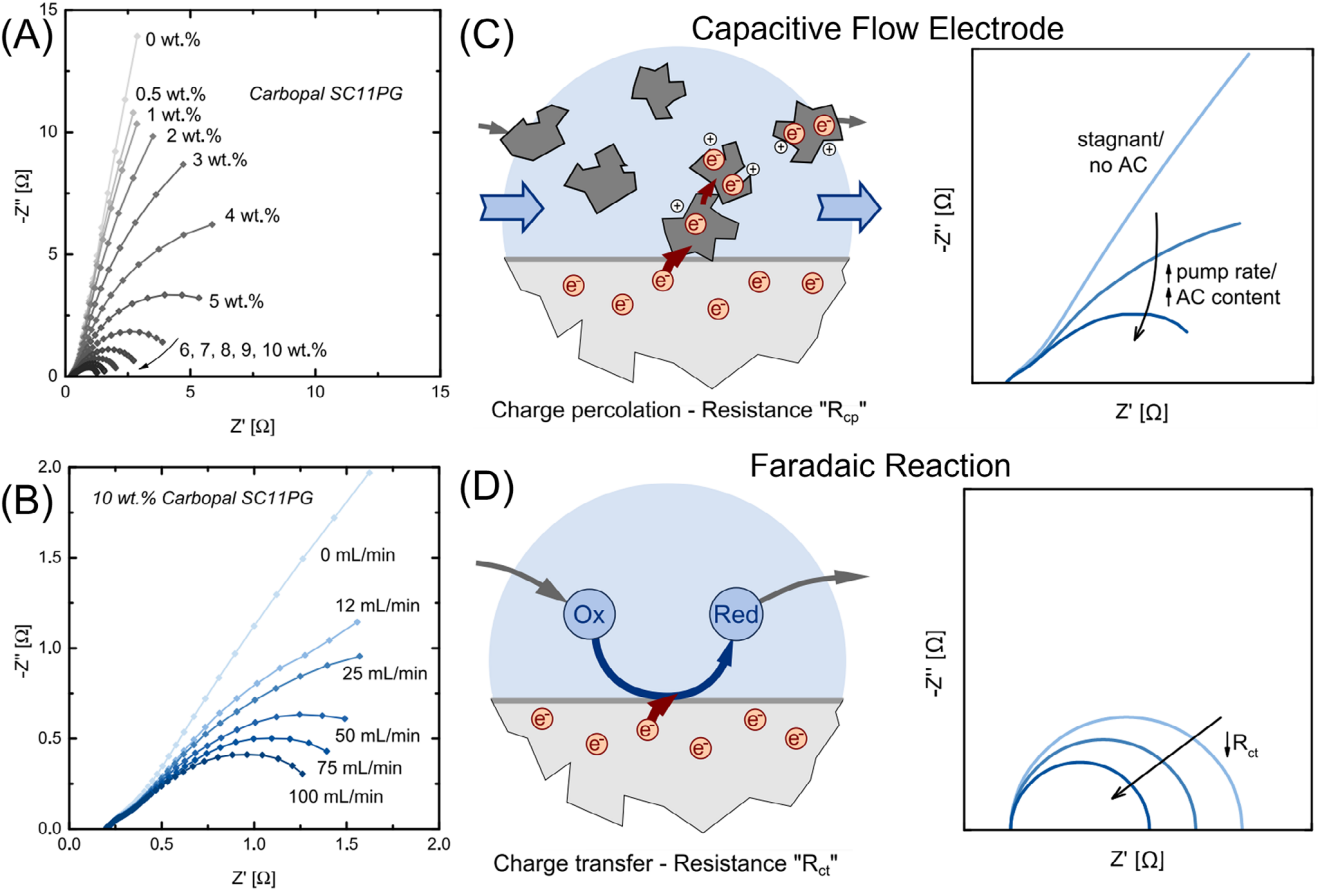
EIS Measurements of the slurry system. A) Variation in the EIS spectra of the activated carbon content with a constant NaCl concentration of 60 g L^−1^ and a flow‐electrode flow rate of 150 mL min^−1^; B) variation of the flow‐electrode flow rate at a carbon loading of 16 wt% and a 60 g L^−1^ NaCl concentration; C) Illustration of the effect of an electric current induced by convection on EIS spectra of a system based on capacitive flow‐electrodes, and comparison to D) an electrochemical system with faradaic reactions. Reprinted with permission from Ref. [12].

In addition to computational approaches, operando electrochemical impedance spectroscopy (EIS) has emerged as a powerful, non‐invasive tool for monitoring the real‐time evolution of the electro‐percolation network.^[^ ^2^, ^4^, ^19^ ^]^ This is reflected by the change from high resistance to well‐defined semicircles at lower impedance values beyond the percolation threshold in Figure 4A.^[^ ^12^ ^]^ Only a small shoulder is seen in the plot at high frequencies, together with a straight line at an angle of around 80° toward the low frequencies in particle‐free solution. A slightly depressed semicircle develops in the lower frequency range with the addition of activated carbon, which becomes even smaller with increasing concentrations, resulting from an increasing capacitance and a decreasing interfacial resistance within the flow electrode. Similarly, the influence of flow rate could be experimentally validated by operando EIS in Figure 4B, where an improved charge percolation is observed at higher flow rates. Notably, a distinguishing feature of EIS in slurry systems lies in its ability to resolve the electrochemical processes associated with different mechanisms. A good example would be the origin of two distinct resistances in the low‐frequency regions. As shown in Figure 4C,D the charge percolation resistance (R_CP_) arises from electron transport through conductive networks in capacitive flow electrodes, which will be enhanced by higher solid contents and flow‐induced charge removal in the FCDI systems. In contrast, the charge transfer resistance (R_CT_) reflects interfacial electron‐transfer kinetics during faradaic reactions (such as OER) in the fluidized electrolysis.^[^ ^5^, ^12^ ^]^ Therefore, the integration of operando EIS and computational modeling could provide a more complete picture of the electro‐percolation network in the slurry systems.

## 4 Advanced Characterization Techniques in Slurry Electrolysis

The complexity of slurry systems demands advanced characterization techniques that capture the transient behavior of particulates in real time.^[^ ^4^ ^]^ While traditional methods are still valuable, they are mostly developed for homogeneous systems and cannot handle slurry systems due to their intermittent particle contacts and evolving microstructures. Another challenge lies in the metastable hydrodynamic behavior of the slurry, which strongly depends on the solid content and time. To this end, several advanced techniques have been developed and adapted for slurry systems, including advanced rheology, particle electrochemistry, and in situ imaging.^[^ ^13^, ^15^, ^16^, ^32^ ^]^

**Figure 5:**
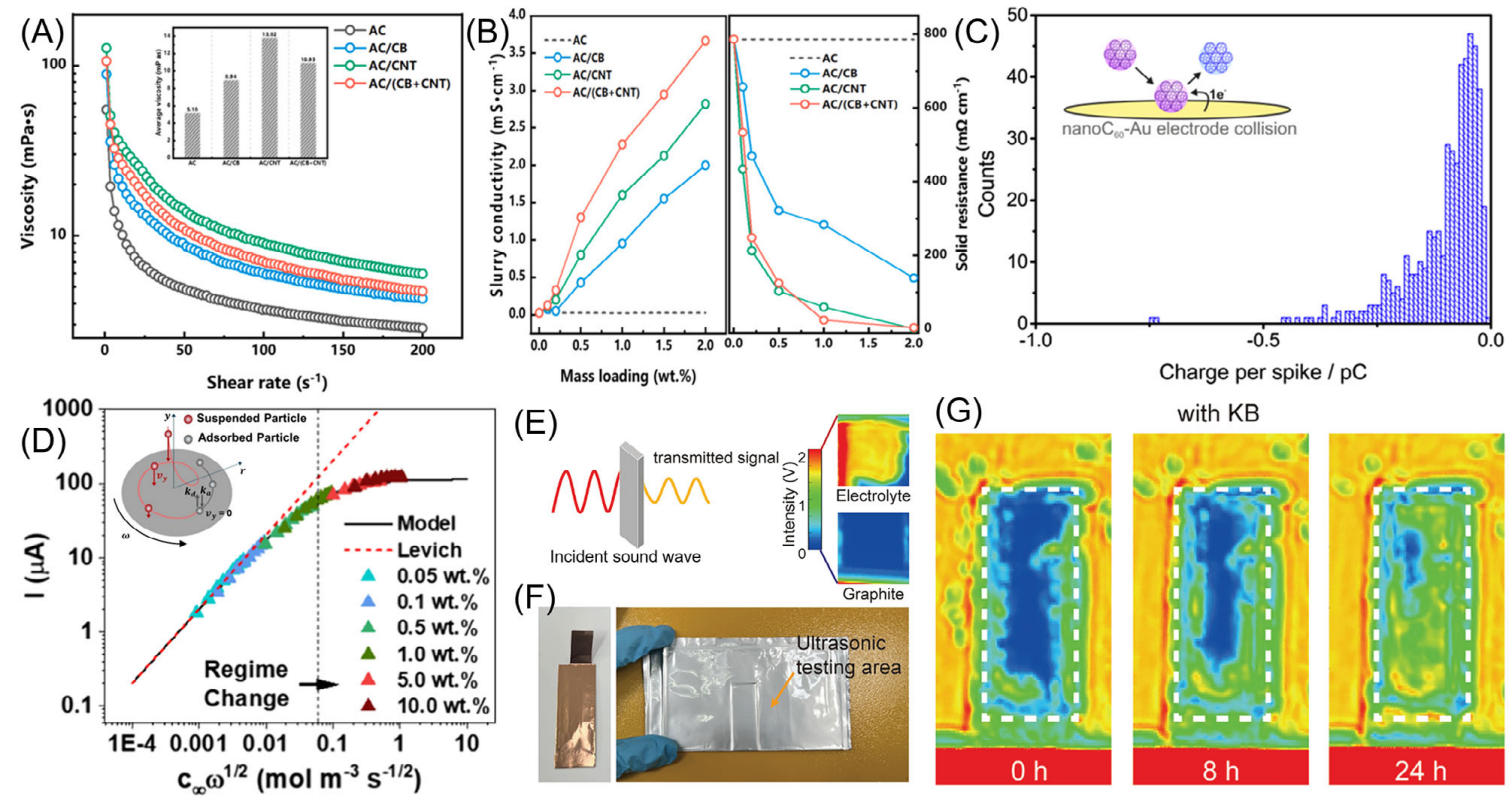
Advanced characterization techniques in slurry electrolysis. A) Rheological measurements, B) the slurry conductivity of four slurries, and film resistivity after freeze‐drying slurry with different total content of conductive agents from 0.1 to 2.0 wt%; C) charge distribution with a bin size of 0.01 pC for the one‐electron reduction of C_60_ nanoparticles impacting a gold microelectrode in buffered acetonitrile; D) Levich and particle self‐crowding regimes from rotating disk electrode (RDE) measurements. Inset shows the nondissolvable particle electrochemistry model; E)–G) in situ ultrasonic imaging of the semi‐solid graphite anodes: E) working principle of the ultrasonic imaging experiments, F) digital photograph showing the aluminum‐plastic bag used, and G) the collected ultrasonic images after 0, 8, and 24 h resting of the graphite anode slurries of with KB as the conductive additives. A), B) Reprinted with permission from Ref. [13]. C) Reprinted with permission from Ref. [16]. D) Adapted with permission from Ref. [15]. E)–G) Reprinted with permission from Ref. [32].

The rheological behavior is an important aspect for slurry electrodes because it indicates flowability and hydrodynamic stability.^[^ ^33^ ^]^ Specifically, electro‐rheology has been the gold standard for measuring the properties of slurry in a shear (or dynamic) environment using parallel plate and concentric cylinder geometries. Alternating current (AC) and direct current (DC) measurements can be coupled to these systems by applying an electrical potential across the sample as the inner cone or top plate rotates at varying velocities (shear rates).^[^ ^3^, ^4^ ^]^ Electrical conductivities are extracted as a function of shear rate and voltage from these experiments, which provides direct evidence of forming an electro‐percolation network. For instance, Youssry et al. performed a comprehensive study on how the (electro‐)rheological properties of different suspensions change under exposure to shearing environments.^[^ ^34^ ^]^ As shown in Figure 5A, viscosity measurements across a range of shear rates revealed the flow behavior of different electrode formulations for subsequent system designs and process optimizations. The DC measurements were applied to evaluate the electrical conductivity in the slurry in Figure 5B. As expected, the conductivity in a multi‐component slurry improved more significantly than that of the conventional slurries with the increasing contents due to the formation of new conductive pathways by activating or reviving those “dead” branches.^[^ ^13^ ^]^

Along with the collective information from electro‐rheological measurements, recent advances in impact electrochemistry offer valuable information about the electrochemical response of individual nanoparticles in slurry.^[^ ^17^, ^35^ ^]^ As shown in Figure 5C, Stuart et al. reported the direct electrochemical detection of impacting carbon C_60_ nanoparticles in a non‐aqueous solution. A total of 481 impact spikes were recorded for the one‐electron reduction of impacting nano‐C_60_, and the area under each current‐time spike was integrated to give the charge for the reduction of each impacting nanoparticle in the charge distribution for the impact events.^[^ ^36^ ^]^ Likewise, Zhou et al. employed the single‐particle impact electrochemistry (SPIE) technique to provide a close‐up view of a representative transient current during the collision‐exfoliation process in the fluidized electrochemical exfoliation of layered transition metal dichalcogenides at a single‐particle level.^[^ ^35^ ^]^ Meanwhile, Takeuchi et al. also proposed the use of a rotating‐disk electrode (RDE) technique for modeling particle self‐crowding of nanoparticle suspensions.^[^ ^15^ ^]^ As shown in Figure 5D, the interfacial phenomena associated with the finite size of the charge carrier were taken into account by considering diffusion and hydrodynamic transport toward the electrode surface, and the kinetics of physical adsorption and desorption in the RDE experiments. At lower concentrations, current scales linearly with *c_∞_ω^1/2^* as indicated in the Levich equation (*c_∞_* is the particle concentration in the bulk solution and *ω* is the angular rotation speed). Above a certain concentration and rotation rate threshold, the electrochemistry deviates from traditional solution theory with a maximum attainable current due to particle “self‐crowding” where reacted particles on the electrode surface reduce the area accessible for charge transfer by unreacted particles.^[^ ^15^ ^]^ This offers a better understanding of the hydrodynamic electrochemistry of solid suspensions under nonideal conditions.

Finally, the visualization of the slurry behavior via in situ imaging techniques would be one of the most powerful approaches for slurry systems.^[^ ^32^ ^]^ As shown in Figure 5E–G, Li et al. developed an in situ ultrasonic imaging technique to investigate the electrolyte distribution and graphite‐particle aggregation in SSFBs.^[^ ^32^ ^]^ The blue color in the color scale in the ultrasonic images indicates low transmission due to more solid materials (i.e., graphite particles), while the red color is attributed to the presence of the liquid electrolyte. It is found that the semi‐solid graphite anodes containing Ketjen black (KB) are more difficult to homogenize due to particle agglomeration on cessation of shear. In contrast, the graphite anode slurry without KB becomes more uniform in a much shorter period of time.^[^ ^32^ ^]^ Apart from this, microscopic imaging and X‐ray microtomography (micro‐CT) also provide the 3D particle distribution of the slurry at different spatial resolutions.^[^ ^8^, ^37^ ^]^

## 5 Perspectives and Conclusion

**Figure 6:**
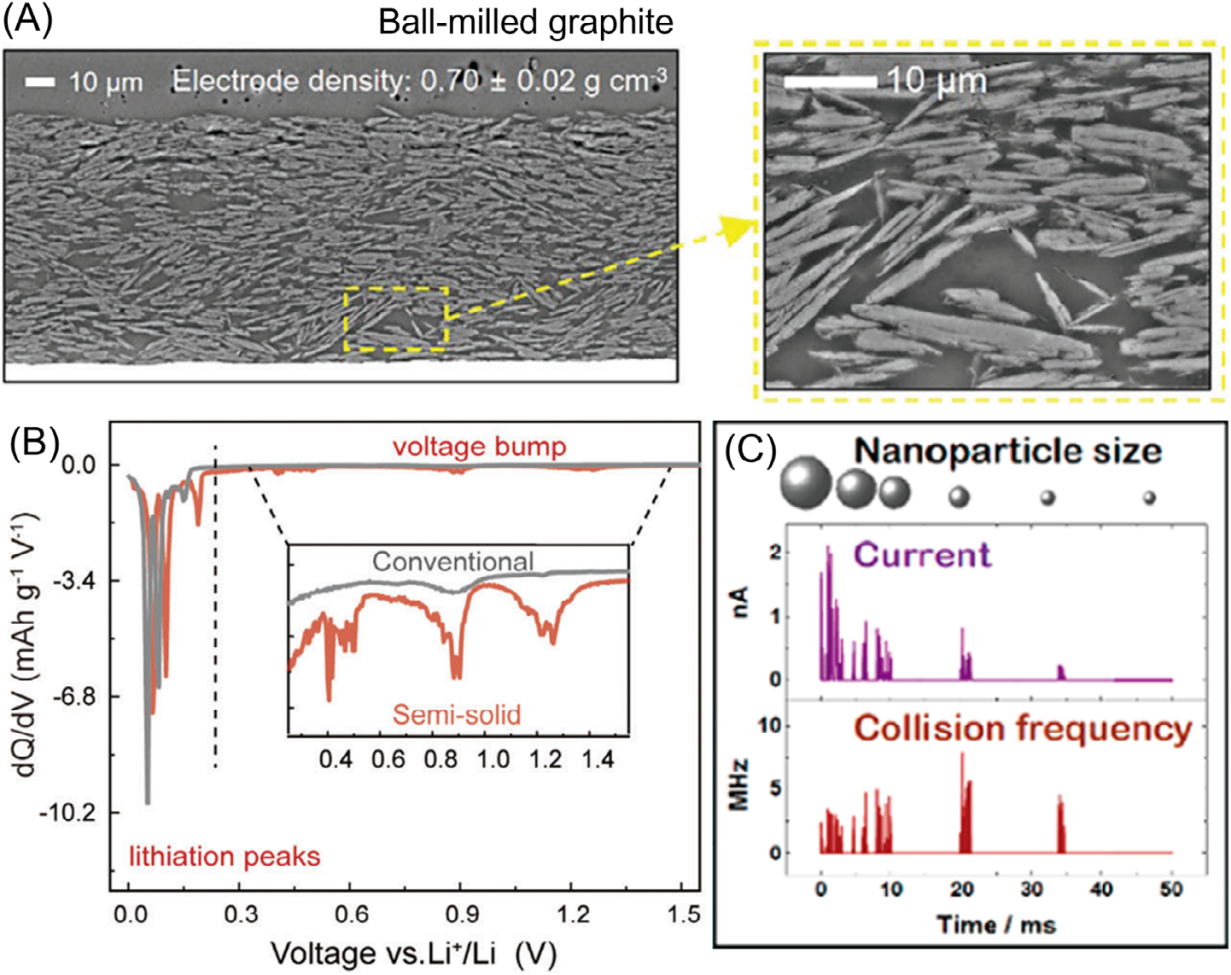
Challenges in slurry electrolysis. A) Representative SEM images of the cross‐section of anodes made of ball‐milled. The magnified image shows the house‐of‐card structure, made up of the plate‐like ball‐milled graphite particles, including the pyramid‐shaped hollow inside; B) comparison of the differential capacity curves (d_Q_/d_V_), showing additional reduction peaks in the case of KB‐containing semi‐solid graphite anode; C) the collision frequency of a single Ag nanoparticle at the electrode and the corresponding current response. A) Reprinted with permission from Ref. [29]. B) Reprinted with permission from Ref. [32]. C) Adapted with permission from Ref. [38].

Despite its advantages, slurry electrolysis introduces a unique set of challenges that stem from its evolving microstructure.^[^ ^4^ ^]^ One of the most persistent issues would be the instability of the slurry and electrode architecture. As shown in Figure 6A, the semi‐solid electrode employing ball‐milled graphite or similar materials could adopt a “house‐of‐cards” microstructure in the SEM images by stacking plate‐like particles in the slurry, which introduces porosity and compromises the slurry stability during repeated charge‐discharge cycles.^[^ ^29^ ^]^ Unwanted side reactions may also occur, particularly in the slurry systems with conductive additives such as Ketjen black. This reduces the Coulombic efficiency and accelerates electrode degradation.^[^ ^32^ ^]^ The intermittent contacts between flowing particles also lead to fluctuating electrochemical signals in Figure 6B, thus having an adverse effect on the reliability and reproducibility. Such problems become more severe when taking the particle size distribution into account. As shown in Figure 6C, the Ag nanoparticles collide more frequently with the electrode at higher current densities and with smaller particle sizes. Hundreds to thousands of nanosecond‐scale collisions may occur when a particle interacts with the electrode before it diffuses back into the solution, making it very complicated for experimental and theoretical modeling.^[^ ^38^ ^]^ Finally, the practical use of slurry electrolysis requires a delicate balance between solid content (i.e., energy density or conductivity) and flowability of the slurry. Uneven distribution and clogging could occur for slurries with unsatisfactory rheological behavior. For a productive slurry electrolysis, longer continuous runs should be conducted to test for the occurrence of clogging, which may appear due to the unexpected, prolonged buildup of solids.^[^ ^30^ ^]^ Meanwhile, the spent carbon additives in slurry electrolysis could be recovered by promising direct recycling approaches. For instance, mechanical separation, such as filtration or centrifugation, can effectively separate carbon particles from the electrolyte slurry, while solvent‐based regeneration approaches help restore the electrochemical performance of carbon additives by removing surface impurities.^[^ ^3^, ^4^ ^]^

Finally, we provide a concise outlook for future slurry electrolysis: i) performance metrics and standardized protocols that capture activity, energy efficiency, and durability should be established across diverse applications; ii) design new materials that enable a stable electro‐percolation network, particularly at a low percolation threshold, while maintaining flowability by optimizing the particle morphology and size distribution. This may involve tailoring surface chemistries and incorporating mediator‐enabled charge transfer; iii) develop operando and inline diagnostics that couple high‐bandwidth electroanalysis with flow‐resolved imaging, rheological probes, and computational CFD–DEM modeling to correlate the structure‐performance correlation and to predict fouling; and iv) system‐level strategies that encompass modular reactor architectures, rigorous gas‐liquid‐solid management, and closed‐loop recovery and regeneration of solids. This requires an interdisciplinary approach that integrates material science, electrochemistry, and system‐level engineering to fully realize the practical potential of slurry electrolysis.^[^ ^3^, ^8^ ^]^

## Author Contributions

All authors drafted, discussed, and commented on the manuscript.

## Conflict of Interests

The authors declare no conflict of interest.

## Terminology

Slurry is usually referred to a particle suspension in dilute to semi‑concentrated regime with near‐Newtonian to shear‐thinning rheology. In the dilute region (volume fraction, φ ≤ 5%), solid particles are well separated, where hydrodynamics is continuum‐dominated. Charge transfer is dominated by ionic conduction through the electrolyte. The electronic contact between particles and the current collector is transient and collision‐mediated. In the semi‑concentrated regime (φ ≈ 5 ∼ 20%), non‐Newtonian behavior (typically shear thinning) occurs due to hydrodynamic interactions and near‐field contacts. Charge transfer is mixed by ionic conduction and short‑lived particle clusters.

Fluidized usually refers to a hydrodynamic operating mode where particles are suspended by flow above the minimum fluidization velocity, with transient, collision‑mediated charge transfer rather than a persistent network.

Semi‑solid is typically referred to a dense, percolated paste (φ ≥ 20%) above the electrical percolation threshold (φc), which exhibits a yield stress and a persistent conductive network. Electronic conduction is dominated by the particle network, while ionic conduction in the interstitial electrolyte provides complementary pathways.

## Data Availability

The data that support the findings of this study are available from the corresponding author upon reasonable request.
